# Widely Used Mutants of *eiger*, Encoding the *Drosophila* Tumor Necrosis Factor, Carry Additional Mutations in the NimrodC1 Phagocytosis Receptor

**DOI:** 10.1534/g3.120.401800

**Published:** 2020-10-30

**Authors:** Albana Kodra, Claire de la Cova, Abigail R. Gerhold, Laura A. Johnston

**Affiliations:** * Department of Genetics & Development, Columbia University Irving Medical Center, Vagelos College of Physicians and Surgeons, New York, NY; † Department of Biological Sciences, University of Wisconsin, Milwaukee, WI; ‡ Department of Biology, McGill University, Montréal, QC

**Keywords:** cell competition, cell death, Eiger/TNF, NimC1 mutations, phagocytosis

## Abstract

The process of apoptosis in epithelia involves activation of caspases, delamination of cells, and degradation of cellular components. Corpses and cellular debris are then rapidly cleared from the tissue by phagocytic blood cells. In studies of the *Drosophila* TNF, Eiger (Egr) and cell death in wing imaginal discs, the epithelial primordia of fly wings, we noticed that dying cells appeared to transiently accumulate in *egr*^*3*^ mutant wing discs, raising the possibility that their phagocytic engulfment by hemocytes was impaired. Further investigation revealed that lymph glands and circulating hemocytes from *egr*^*3*^ mutant larvae were completely devoid of NimC1 staining, a marker of phagocytic hemocytes. Genome sequencing uncovered mutations in the *NimC1* coding region that are predicted to truncate the NimC1 protein before its transmembrane domain, and provide an explanation for the lack of NimC staining. The work that we report here demonstrates the presence of these *NimC1* mutations in the widely used *egr*^*3*^ mutant, its sister allele, *egr*^*1*^, and its parental strain, *Regg1^GS9830^*. As the *egr*^*3*^ and *egr*^*1*^ alleles have been used in numerous studies of immunity and cell death, it may be advisable to re-evaluate their associated phenotypes.

The *Drosophila* genome encodes a single TNF homolog, known as Eiger (Eda-like cell death trigger, Egr) (Igaki *et al.* 2002; Moreno *et al.* 2002; Narasimamurthy *et al.*). Egr is expressed in many different tissues and plays various roles in cellular processes such as the immune response, energy homeostasis, and JNK-dependent cell death. Since its identification, numerous studies on cell death and immunity have utilized the *egr*^*3*^ and *egr*^*1*^ alleles, which were generated by imprecise excisions of the *Regg1^GS983^*^0^ *P* element and resulted in deletions of the first coding exon of the *egr* gene (Igaki *et al.* 2002). Both *egr*^*3*^ and *egr*^*1*^ strains are homozygous viable and considered severe loss-of-function alleles.

Dead cells in *Drosophila* are commonly removed from tissues by phagocytic engulfment by plasmatocytes, the most abundant of the circulating hemocytes in the larva (Abrams *et al.* 1993; Sonnenfeld and Jacobs 1995; Franc *et al.* 1999; Shklyar *et al.* 2013). Plasmatocytes carry cell surface receptors for the recognition and rapid engulfment of bacteria, dead cells and cellular debris, such as Eater (Kocks *et al.* 2005; Chung and Kocks 2011), NimrodC1 (NimC1) (Kurucz *et al.* 2007; Honti *et al.* 2013) and Draper (Manaka *et al.* 2004). A frequently used marker for plasmatocytes in *Drosophila* is positivity for NimC1, a transmembrane protein characterized by the presence of a special type of EGF repeat known as the NIM repeat, located immediately proximal to a conserved CCxGY motif (Somogyi *et al.* 2008). The *NimC1* gene is part of a cluster of four *NimC* (*NimC1-4*) genes in the midst of several other related *Nimrod* genes at 34E on chromosome 2. Nimrod proteins contain 2–16 NIM repeats as well as additional conserved residues at their amino termini. The Nimrod proteins, together with Eater and Draper, form a conserved superfamily of 12 proteins in *Drosophila*, and Nimrod proteins are also encoded in the *C. elegans* and mammalian genomes (Melcarne *et al.* 2019a). Loss of any Nimrod protein diminishes the capacity of hemocytes to fight microbes. For example, RNAi against *NimC1* has implicated it in bacterial phagocytosis (Kurucz *et al.* 2007), while complete loss of *NimC1* demonstrated cooperativity between NimC1 and Eater in the recognition and phagocytosis of bacteria (Melcarne *et al.* 2019b). In addition, Egr has been reported to have a role in regulating phagocytosis of certain bacteria (Schneider *et al.,* 2007). Here we report that *egr*^*1*^ and *egr*^*3*^, two commonly used *egr* alleles, and their parental strain *Regg1^GS9830^*, carry additional mutations in the *NimC1* phagocytosis receptor gene.

## Materials and Methods

### Drosophila genetics and husbandry

Flies were raised at 25° on standard cornmeal-molasses food supplemented with fresh dry yeast. The following strains were used: *egr*^*1*^*, egr^3^* and *egrRegg*^*1*^ (Igaki *et al.* 2002), *egr*^*31*^ (gift of H. Kanda), *egr*^*3AG*^ and *egr*^*1AG*^ (generated in this work), *HmlΔ-DsRed^nls^/CyO* (gift of K. Brückner; (Makhijani *et al.* 2011)), *yw hsflp^122^* (gift of G. Struhl), *act > y+^STOP^ > Gal4* and *tub > Myc*^*STOP*^* > Gal4* (de la Cova *et al.* 2004), *OregonR* (Bloomington Drosophila Stock Center).

### Cell death assays

Eggs from appropriate crosses were collected on yeasted grape plates for 2-4 hr and allowed to develop at 25° in a humid chamber for 24 hr. Prior to collecting the eggs, two 30 min pre-collections were carried out to allow females to void any developing eggs. At hatching, larvae were transferred to food vials supplemented with fresh yeast paste at densities of less than 50 larvae/vial to prevent crowding. To generate ‘loser’ clones in a competitive context, a *tub > myc, y+*^*STOP*^* > Gal4* cassette (> represents a FLP-recognition target (FRT) site) was used to excise the >*myc, y+*^*STOP*^ cassette and generate *tub > Gal4, UAS-GFP* expressing “loser” cells in WT and in *egr*^*3*^ mutants. FLP recombinase, under heat shock (HS) control, was activated by HS of larvae in a 37° water bath for 10 min, at 48 hr after egg laying (AEL). Post-HS, larvae were allowed to develop at 25° for 24 or 48 hr. To generate Myc-expressing clones, *act > y+^STOP^ > Gal4* was used to generate *act > Gal* clones that expressed UAS-GFP and UAS-Myc in WT and in *egr*^*3*^ mutants. *act > Gal4* clones were induced in larvae with a HS in a 37° water bath for 6 min. Clones were allowed to grow in the wing discs for 24 or 48 hr, as described (de la Cova *et al.* 2004; Meyer *et al.* 2014; Alpar *et al.* 2018). The wing discs were then dissected from larvae at the indicated times after clone induction (ACI). A detailed protocol is available upon request.

### Larval dissection and imaging

Wing imaginal discs were dissected from third instar larvae as indicated above, and fixed in 4% paraformaldehyde in phosphate-buffered saline (PF-PBS) for 20 min at room temperature, and washed 3-5 times for 20 min each with 0.01% Tween-20 in PBS (PBTw). Larval carcasses were stained with Rabbit anti-Cleaved Caspase-3 (Cas-3) at 1:100 (Cell Signaling Technology, cat. # 9661). Secondary Alexa Fluor 555 Goat anti-Rabbit IgG antibodies (1:600) were purchased from Molecular Probes (cat.# A-21429). Lymph glands were stained with plasmatocyte-specific P1 antibodies (Mouse anti-NimC; 1:100) (Kurucz *et al.* 2007) and subsequently, Alexa Fluor 555 (Invitrogen) secondary antibodies. Hoechst 33258 (Sigma) was used to stain DNA. Wing discs and lymph glands were mounted in VectaShield Antifade (Vector Laboratories, Cat# H-1000) on glass slides. Images were acquired with a Zeiss Axiophot microscope with Apotome and processed using ImageJ and Adobe Photoshop.

### Hemocyte immunohistochemistry and image processing

Hemocytes were collected from 10-20 experimental larvae, by bleeding from a small tear in the posterior cuticle into a 10-fold volume of PBS. Cells were then transferred to a coverslip and allowed to settle for 30 min at room temperature in a humidity chamber. All subsequent steps were performed directly on the coverslip. Cells were fixed in 4% PFA in PBS for 7 min at room temperature, washed 3 times in PBS, permeablized for 5 min in 0.1% Triton in PBS (PBT), blocked for 5 min in 10% normal goat serum (NGS) in PBT and then incubated with primary antibody in 10% NGS in PBT either overnight at 4° or for 30 min at room temperature. Washes were carried out in PBT and secondary incubation was performed for 30 min at room temperature in 10% NGS in PBT. Cells were then washed 2 times in PBT, followed by 2 washes in PBS. A final 5 min incubation with DAPI in PBS was performed. Coverslips were mounted in Slowfade Gold Antifade (Molecular Probes, cat # S36937). Primary antibodies used were plasmatocyte-specific Mouse anti-P1 (1:100) (Kurucz *et al.* 2007). Secondary antibodies were Alexa Fluor 555 (1:500, Invitrogen). Images were collected on a Zeiss Axio Imager M1 and were processed using ImageJ and Adobe Photoshop.

### Sequencing of egr mutant strains

Genomic DNA was isolated from homozygous adult female flies. 100 ng of DNA was amplified using the primer sets as described in Honti *et al.* 2013. The 355bp deletion, 5bp insertion and 6bp microdeletion were found in the fragment amplified by P11189fw (CGCAGGAGCCTACGATAATC) and P11189rev (AAGGAATGTGGACACCATAG). The fragments were cloned into a pCR4-TOPO-TA vector for sequencing using the common sequencing primers M13. Primer sequences are listed in Supplementary Table 3.

### Outcrossing and genotyping of egr alleles

*egr*^*1*^* and egr^3^* alleles (hereafter *egr*) were treated identically. *egr* mutant virgin females were crossed to *OregonR* (*OreR*) males and the resulting F1 heterozygous *egr**/+* virgin females were backcrossed to *OreR* males. Ten F2 males (either *egr**/+* or +/+) were singly crossed to virgin *OreR* females, killed and used for single-fly PCR (Gloor *et al.* 1993) to identify males carrying the *egr* alleles, but no longer carrying the 355bp deletion in *NimC1*. A single *egr*-positive, *NimC1*-negative line for each allele was carried forward by crossing F3 virgin females back to *OreR* males. This process was repeated four times, after which *egr* alleles were re-isolated by crossing to *+; Sco/CyO actin-GFP*, a chromosome II marker/balancer strain similarly crossed into an *OreR* background. These alleles were named *egr*^*1AG*^ and *egr*^*3AG*^. Genotyping of *egr*^*1*^ and *egr*^*3*^ used primers flanking the reported deletion in each strain: Egr_F1 (CCAGAGCCCACTGTATCACC) and Egr_R3 (TCACCTCCTTTTGGAACTCG) amplify a ∼1500bp and ∼2000bp fragment in *egr*^*1*^ and *egr*^*3*^*,* respectively. Genotyping of *NimC1* used primers flanking the 355bp deletion and 5bp insertion between nucleotides 1582 to 1937 (Honti genome annotation). Nimrod_del_F1 (CCGGGCTACGTAATGAGAAA) and Nimrod_del_R1 (CAATTTGAGTGCGGAACCTC) amplify a 656bp fragment in WT animals and a ∼300bp fragment in animals bearing the *NimC1* deletion. Primer sequences are listed in Supplementary Table 3.

### Statistical analysis

T-tests were carried out using two tails of unequal variance.

### Data availability

**Figure 1:**
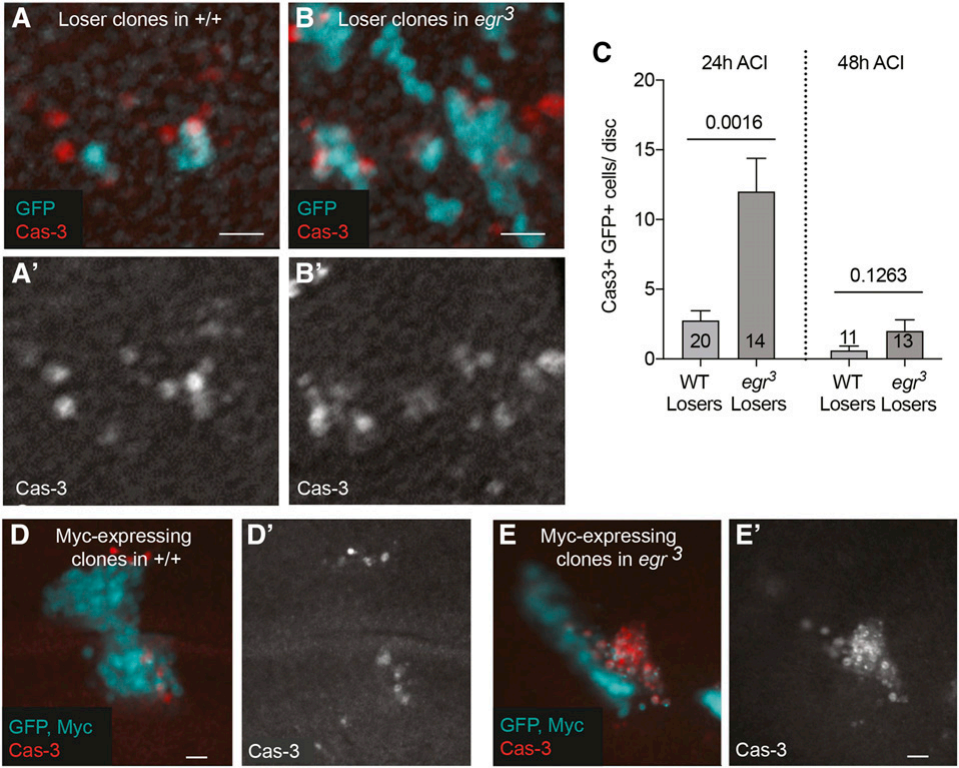
Dying cells transiently accumulate in *egr*^*3*^ mutants. A. Clones in A and B were examined 24 hr after clone induction (ACI). Clones of loser cells in a wildtype (WT) wing disc, expressing GFP (cyan) and cleaved caspase 3 (Cas-3); Cas-3 channel is shown in A’. Larval genotype: *yw hsflp/w;; tub > myc, y+^STOP^ > Gal4, UAS-GFP/+.* Genotype of the WT loser clones: *yw hsflp/w;; tub > Gal4, UAS-GFP/+.* B. Loser clones, expressing GFP and Cas-3, in a *egr*^*3*^ mutant wing disc; Cas-3 channel is shown in B’. Larval genotype: *yw hsflp/w; egr*^*3*^*/egr^3^; tub > myc, y+^STOP^ > Gal4, UAS-GFP/+.* Genotype of these loser clones: *yw hsflp/w; egr*^*3*^*/egr^3^; tub > Gal4, UAS-GFP/+.* C. Quantification Cas-3-positive, GFP-positive (loser) cells per disc at 24hr ACI from A-B, and from the same genotypes (WT or *egr*^3^) at 48 hr ACI. Numbers of wing discs examined for each genotype is shown in the bars. P values were determined using 2-tailed ttests with unequal variance. D. Image of a Myc-expressing clone, marked by expression of GFP (cyan), in a wildtype wing disc. Cas-3 positive cells are shown in red. D’ shows Cas-3 as a single channel. Larval genotype: *yw hsflp/w; act > CD2^STOP^ > Gal4, UAS-GFP/UAS-Myc.* Clone genotype: *yw hsflp/w;; act > Gal4, UAS-GFP/UAS-Myc.* E. Image of a Myc-expressing clone, marked by expression of GFP (cyan), in an *egr*^*3*^ mutant wing disc. Cas-3 positive cells are shown in red. E’ shows Cas-3 as a single channel. Larval genotype: *yw hsflp/w; egr*^*3*^*/ egr^3^; act > CD2^STOP^ > Gal4, UAS-GFP/UAS-Myc*. Clone genotype: *yw hsflp/w; egr*^*3*^*/ egr^3^; act > Gal4, UAS-GFP/UAS-Myc.* Clones in D and E were examined 48 hr ACI. Scale bars represent relative size.

**Figure 2:**
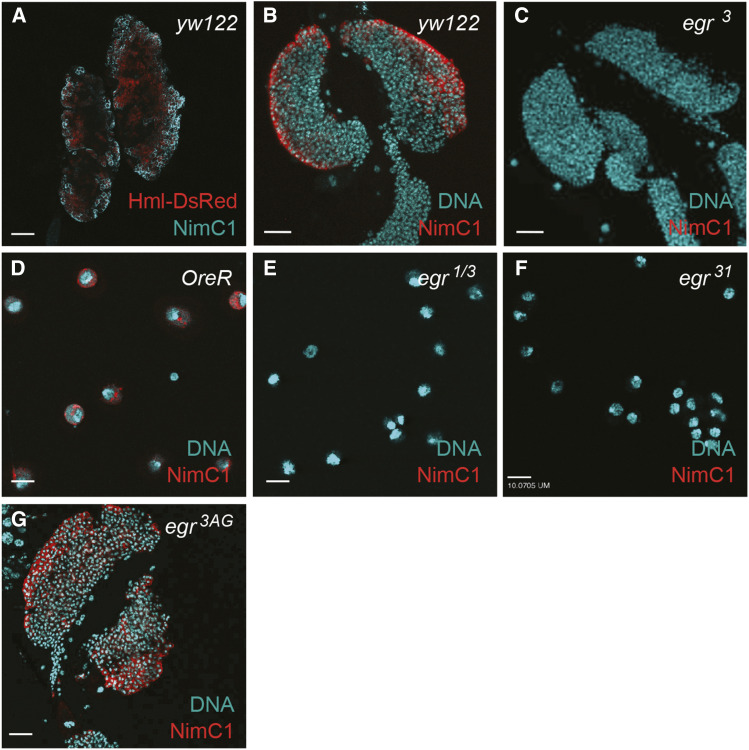
Lymph glands from *egr*^*3*^ mutant larvae and circulating hemocytes from *egr*^*1/3*^ and *egr*^*31*^ mutant larvae are negative for NimC1 staining. A. HmlΔ-DsRed (red) and NimC1 (green) are expressed in many hemocytes in lymph glands from larvae of the genotype *ywhsflp122; HmlΔ-DsRed^nls^.* The *ywhsflp122 (*abv. *yw122)* strain served as a wildtype control. Scale bars in A-C and G represent 50 um. B. NimC1 (red) staining in the primary lobes of lymph glands from control larvae. C. Lymph glands from *egr*^*3*^ mutant larvae have no NimC1-positive hemocytes. D. Circulating hemocytes from the hemolymph of *OreR* control larvae stain positively for NimC1 (red). Scale bar represents 10um. E-F. Circulating hemocytes from *egr*^*1/3*^ transheterozygous larvae (E) and from *egr*^*31*^ mutant larvae (F) lack positivity for NimC1. Scale bars represent 10um. G. NimC1 staining (red) in the *egr*^*3AG*^ mutant lymph glands, in which the *NimC1* locus was restored to WT.

**Figure 3:**
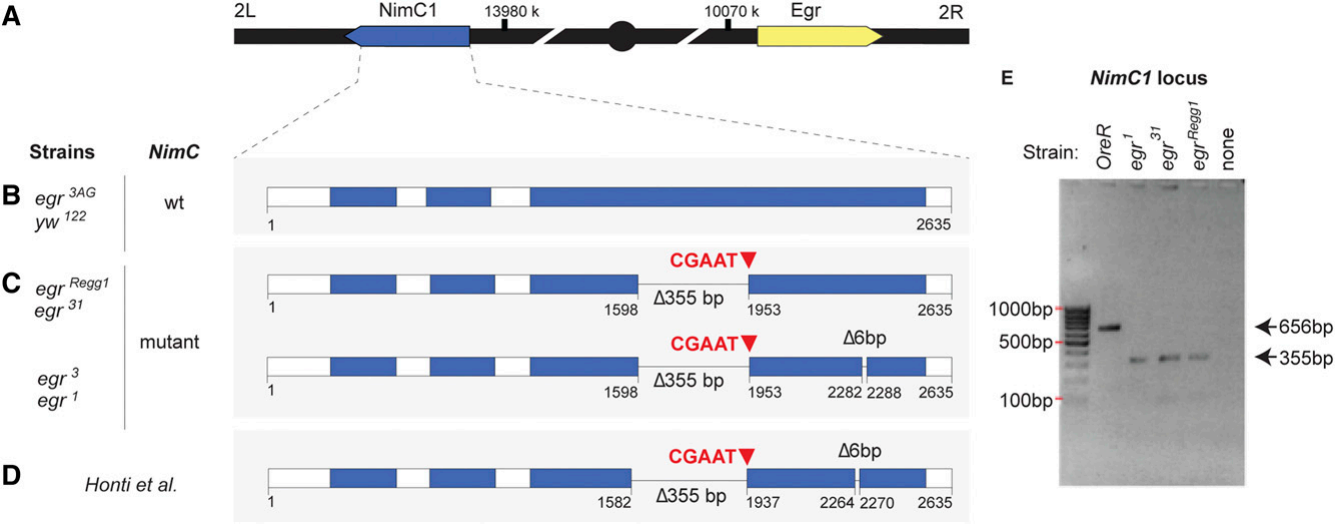
Summary scheme of mutations in the *NimC1* locus in various *egr* mutants. A. Schematic representation of Chromosome 2, where the *NimC1* locus is located at on the left arm (2L) and the *egr* gene on the right arm (2R). B. Representation of the *NimC1* locus from *yw122* flies, used as a WT strain. This sequence, and that from the outcrossed *egr*^*3AG*^ strain, is identical to the reference genome (*D. melanogaster* version r5.23). PCR genotyping suggests that the *OreR* WT strain is also wild type at the *NimC1* locus. Numbering as in https://flybase.org/decoratedfasta/FBgn0259896. C. Representation of the *NimC1* locus in the *egr*^*31*^ precise excision allele, the parental allele *egr*^*Regg*^^*1*^, and the *egr*^*1*^ and *egr*^*3*^ daughter strains. Two deletions, of 355 bp and 6bp, and an insertion of 5 bp, were found in *egr*^*1*^ and *egr*^*3*^. PCR genotyping in *egr*^*31*^ and the parental strain, *egr*^*Regg*^^*1*^ indicates that they also carry the 355 bp deletion (E); although not notated here, it is likely that they also carry the 5 bp insertion and 6 bp deletion. D. Representation of the *NimC1* locus from Honti *et al.,* 2013. Note that the locus numbering is slightly different than in B-D, presumably due to an earlier genome annotation. The mutations are identical to those found in *egr*^*1*^ and *egr*^*3*^ and similar to *egr*^*31*^ and the parental line, *egr*^*Regg*^^*1*^ (C). E. Gel electrophoresis of the results of PCR genotyping of *NimC1* in the *egr* mutants indicated at top, using primers flanking the 355 bp deletion and 5 bp insertion between nucleotides 1582 to 1937 (Honti *et al.* genome annotation). Primer sequences are listed in Supplementary Table 3 and included in the Reagent Table.

Strains and plasmids are available upon request. Supplementary Table 1 lists genotypes and sources of all *Drosophila* strains and sources and identifiers of other reagents that were used. Supplementary Table 2 lists the genotypes used in Figures 1, 2 and 3. Supplementary Table 3 contains sequences for the primers used in the study. All of this information is also included in the Reagent Table.

## Results

In the course of studying the role of Egr in cell competition, where apoptosis is induced in so-called “loser” cells, we found that dying cells appeared to accumulate in wing discs from *egr*^*3*^ mutant larvae (Figure 1A-E). That cell death was still induced in *egr*^*3*^ mutant loser cells suggested that Egr is not required for the cells to die under the two conditions we examined. However, because dead cells are typically cleared within 2-4 hr from wild-type wing imaginal disc epithelia (Milan *et al.* 1997), the accumulation of Cas-3 positive loser cells that we observed in *egr*^*3*^ mutants suggested that loss of *egr* might impair corpse clearance. This prompted us to examine plasmatocytes in the lymph glands, the major larval hematopoietic organ, from WT and *egr*^*3*^ mutant larvae. We immunostained the lymph glands from both genotypes with anti-NimC1 antibodies, a mixture of P1a and P1b antibodies that specifically recognizes the phagocytic plasmatocytes of the larva (Kurucz *et al.* 2007). As a control, we also examined larvae that carried *HmlΔ-DsRed*, consisting of a hemocyte-specific enhancer/promoter from the *Hemolectin* gene fused to red fluorescent protein (DsRed) that identifies larval hemocytes (Makhijani *et al.* 2011) (Figure 2A). NimC1 is expressed at high levels on the plasma membrane of numerous cells in the primary lymph gland lobes from WT controls, and anti-NimC1 staining overlapped with many HmlΔ-DsRed positive cells (Figure 2A, B). Strikingly, however, no NimC1 positive cells were evident in lymph glands from *egr*^*3*^ larvae (Figure 2C). As the *egr*^*3*^ and *egr*^*1*^ alleles were derived from the same parental strain (Igaki *et al.* 2002), we also tested lymph glands from *egr*^*1*^ larvae, and again found no detectable NimC1 expression (data not shown). To examine circulating plasmatocytes, we isolated hemocytes from larval hemolymph. Although NimC1 was readily observed in circulating hemocytes from *OregonR* (*OreR*) controls (Figure 2D), we detected no NimC1-positive hemocytes in the hemolymph from *egr*^*3*^*/egr^1^* transheterozygous larvae, or from *egr*^*31*^ larvae (Figure 2E-F). *egr*^*31*^ is a precise excision of the *Regg1^GS983^*^0^ *P*-element present in the parental strain. Thus all of the *egr* alleles derived from the *Regg1^GS983^* strain lacked circulating and lymph gland resident plasmatocytes that expressed NimC1.

Honti and colleagues reported that several *Drosophila* strains that were negative for NimC1 staining carried mutations in the *NimC1* gene, which they postulated were scars of mobile element mobilization (Honti *et al.* 2013). Genomic sequencing of these P1-negative strains identified two independent micro-deletions in the *NimC1* gene, including a 6 bp deletion between nucleotides 2264 to 2270 (Honti *et al.* 2013). Another deletion of 355 bp was found between nucleotides 1582 to 1937, accompanied by a 5 bp insertion. Together, Honti *et al.* 2013 found that the 355 bp deletion and the 5 bp insertion generated a frameshift mutation in both the *NimC1** RA* and *RB* transcripts, resulting in new sequences and a premature stop codon (Honti *et al.* 2013). The alterations were predicted to give rise to a truncated NimC1 protein that lacks the intracellular and transmembrane domains and four extracellular NIM repeats, which would account for its absence on the plasma membrane of hemocytes (Honti *et al.* 2013).

To determine whether the *egr*^*1*^ and *egr*^*3*^ mutants carried mutations at the *NimC1* locus, we carried out genomic sequencing of a 1254 bp region that encompasses most of the *NimC1* open reading frame (Figure 3). Our data shows that both *egr*^*1*^ and *egr*^*3*^ contain identical microdeletions and insertions within the *NimC1* gene, consistent with their common parental origin. Each mutant strain has the same 355 bp deletion, 5 bp micro-insertion, and 6 bp micro-deletion described by Honti *et al.* 2013 at residues 2264-2270 (Figure 3C). In addition, using primers flanking the larger, 355 bp deletion in PCR reactions, we found that both the *Regg1^GS9830^* and *egr*^*31*^ strains carried similar lesions (Figure 3E). Since these *egr* mutants were both NimC1 negative (Figure 2E, F), they very likely also carry the premature stop codon generated by the 355 bp deletion and 5 bp insertion. Altogether, these results suggest that these *NimC1* polymorphisms were present in the parental strain (Figure 3C).

To restore the wild-type NimC1 locus to the *egr* mutants, we outcrossed both *egr*^*1*^ and *egr*^*3*^ to the *OreR* wild-type strain and isolated recombinants with the WT *NimC*1 locus and either the *egr*^*1*^or *egr*^*3*^ mutation (see Methods). We then sequenced the *NimC1* locus in these outcrossed *egr* alleles (hereafter called *egr*^*1AG*^ and *egr*^*3AG*^) to verify that the recombination removed the mutant sequences. Both the *egr*^*1AG*^ and *egr*^*3AG*^ strains lacked the deletions and micro-insertions that characterized the original *egr*^*1*^ and *egr*^*3*^ alleles (Figure 3B, E). Consistent with the loss of the deletions, hemocytes from the *egr*^*1AG*^ and *egr*^*3AG*^ mutants regained NimC1 positivity (Figure 2G and data not shown).

## Discussion

Our sequencing data confirm that *egr*^*1*^ and *egr*^*3*^ mutants also carry mutations at the *NimC1* locus, similar to those found previously in other *Drosophila* strains (Honti *et al.* 2013). Since the large deletion and micro-insertion in exon 3 of *NimC1* also exist in the original parental line for the *egr*^*1*^ and *egr*^*3*^ alleles, *egr*^*Regg*^^*1*^, and also in *egr*^*31*^, a precise excision of the *Regg1^GS983^*^0^ *P*-element, it is highly likely that the *NimC1* mutations in each of these *egr* alleles are derived from the parental strain. These *NimC1* mutations are recessive (Honti *et al.* 2013), and we speculate that their presence on each *egr* mutant chromosome in our experiments might explain the transient accumulation of dying cells; perhaps they also account for the infection susceptibility found previously in *egr*^*3*^ mutants (Schneider *et al.* 2007). Consistent with our sequencing results, the genetic backgrounds of the *egr*^*1*^ and *egr*^*3*^ alleles were previously noticed to harbor anomalies that led to *egr*-independent susceptibility to infection by Gram-positive bacteria (Narasimamurthy *et al.* 2009). Complete deletion of *NimC1* has been reported to prevent phagocytosis of latex beads or yeast zymosan particles by plasmatocytes (Melcarne *et al.* 2019b), but whether and how phagocytosis of dying cells may be impaired by the *NimC1* mutations we found here remains to be determined. NIM repeats are thought to mediate protein-protein interactions and clustering of receptors is proposed to be key in phagocytic removal of apoptotic cells (Shklyar *et al.* 2013). If the truncated mutant NimC1 proteins are aberrantly secreted into the hemolymph, as predicted (Honti *et al.* 2013), they could interfere with critical NIM interactions. As the *egr*^*1*^* and egr^3^* alleles have been used in numerous studies of immunity and cell death, it may be worthwhile to re-evaluate some of the phenotypes obtained with these alleles.
